# Discovering the Potential of Cannabidiol for Cosmeceutical Development at the Cellular Level

**DOI:** 10.3390/ph18020202

**Published:** 2025-02-02

**Authors:** Natjira Tassaneesuwan, Mattaka Khongkow, Siriyakorn Jansrinual, Pasarat Khongkow

**Affiliations:** 1 Department of Biomedical Sciences and Biomedical Engineering, Faculty of Medicine, Prince of Songkla University, Songkhla 90110, Thailand; 2 Translational Medicine Research Center (TMRC), Faculty of Medicine, Prince of Songkla University, Songkhla 90110, Thailand; 3 National Nanotechnology Centre (NANOTEC), National Science and Technology Development Agency, Pathumthani 12120, Thailand; mattaka@nanotec.or.th

**Keywords:** cannabidiol, CBD, cytotoxicity, bioactivity, anti-aging, skin cells, cosmetics

## Abstract

**Backgrounds:** Cannabidiol (CBD) has been used for the development of extensive cosmeceutical commercial products. However, the safety and unclear bioactivity of CBD are still concerns and need to be examined to assess the impact of CBD on skin cells through cosmeceutical applications, particularly its impact on anti-aging and wound healing activities. **Methods:** In our study, the cytotoxicity of CBD was investigated on keratinocytes and fibroblasts in short-term and long-term treatments using a sulforhodamine B (SRB) assay and a clonogenic assay, respectively. Next, the antioxidant, anti-aging, and wound healing bioactivities of CBD were assessed. Then, we investigated the expression of the related genes. **Results:** Our results show that CBD at low concentrations (0.625–2.5 µg/mL) was not toxic to cells in the short-term treatment and significantly enhanced the growth of keratinocytes and fibroblasts under long-term exposure. Furthermore, CBD exhibited promising cellular bioactivities, including antioxidant and anti-aging activities in keratinocytes and fibroblasts, and it enhanced wound healing in skin cells. Moreover, CBD has affected the expression of skin regenerative genes in fibroblasts via TGF-β, VEGF, and NF-κB expression. In addition, CBD promoted CO1A2 expression, which is related to collagen production. **Conclusions:** Altogether, our findings confirm the promising potential of CBD, showing that it can be applied in various topical cosmeceutical products. However, further studies, including in vivo studies and clinical trials, should be conducted to confirm the safety and long-term effectiveness of CBD on the skin.

## 1. Introduction

Pollution and ultraviolet (UV) radiation are exogenous factors that can activate free radicals or reactive oxygen species (ROS) on the skin, leading to the cellular senescence of skin cells [1]. The induction of cellular aging and the higher ROS level in cells affect oxidative stress in the cells, which induces cellular damage and promotes cell death [2]. In terms of the molecular mechanism of aging, ROS can induce nuclear factor kappa B (NF-κB), a key driver of cellular stress response and an aging-related gene [3]. NF-κB can also promote inflammation and altered cellular communication, which is one of the aging hallmarks [4]. Inflammation can enhance tissue damage and release cytokines to activate the immune response to eliminate impaired cells. Furthermore, NF-kB is related to the senescence-associated secretory phenotype (SASP) and induces the release of many cytokines such as IL-1, IL-1R, IL-6, CXCR2, and TNF-α [3]. These cause a significant decrease in collagen and elastin production on the dermis layer of human skin [1]. Generally, collagen, elastin, and hyaluronic acid are the major components of the dermis layer, which help skin to be supple, smooth, and elastic [5]. Moreover, ROS can induce the melanogenesis process [6]. Overall, ROS are an important factor in triggering the signal of skin aging, causing skin inflammation, wrinkling, pigment spots, and sagging.

Cannabidiol (CBD) is a phytochemical compound in the cannabinoids group that is found in the cannabis plant, also known as hemp or marijuana. Cannabinoids are a class of terpenophenolic compounds, accumulated mainly in the trichome cavity of the female flower [7]. Normally, the major groups of phytocannabinoids in cannabis plants are ∆9-tetrahydrocannabinol (∆9-THC) and cannabidiol (CBD), the latter of which has no psychoactive effects, while THC has psychoactive effects [8]. Phytocannabinoids can interact with the cannabinoid receptors in the body, known as the endocannabinoid system (ECS). Cannabinoid receptors can be found in the central and peripheral nervous system and can be classified into two types: the CB1 and CB2 receptors [8]. The CB1 receptor is expressed in the brain and central nervous system (CNS), while the CB2 receptor is expressed in immune cells, the peripheral nervous system, testes, and retina [9].

Interestingly, skin possesses its own endocannabinoid system, which plays an important role in regulating cell proliferation, maintaining the skin barrier, and modulating inflammatory processes in the skin via the regulation of the CB1 and CB2 receptors located in the ECM [10]. Previous studies mentioned that CBD has a low binding affinity with CB1 and CB2 and acts as an antagonist at the CB1 receptor [11]. Additionally, CBD functions as a partial agonist of the CB2 receptor and can interact with non-cannabinoid receptors [12]. These include transient receptor potential (TRP) receptors and peroxisome proliferator-activated receptors (PPARs), which are found in various skin cells and influence numerous biological processes [10,13]. CB2 receptor activation has demonstrated potential in inhibiting inflammatory responses and reducing pro-inflammatory cytokines [14]. Moreover, studies involving the genetic deletion of CB1 receptors in mice have shown a decrease in the subdermal fat layer, a characteristic associated with aging [15]. CBD can inhibit the activity of the caspase-1 enzyme via direct binding to the caspase-1 protein, resulting in decreased pyroptosis and apoptosis in human skin keratinocytes from stimulation by H_2_O_2_ [16]. In addition, CB2 receptors can be expressed during the wound healing process, and CB2 agonists were found to decrease the inflammatory response in a mouse model of wound healing, promoting re-epithelization [17]. Thus, the endocannabinoid system significantly influences skin processes, particularly inflammation, which is closely related to aging and wound healing.

Besides interacting with the endocannabinoid system, CBD could reduce the mitochondria-generated ROS in keratinocytes [16] and decreased the levels of 4-HNE and MDA–protein in 2D and 3D fibroblast cell models, which are associated with the antioxidant response and inflammation [18]. Moreover, CBD decreased the expression of pro-inflammatory proteins, including the TNF-α/NF-κB and IκBKB complex in keratinocytes [19], and regulated the inflammatory response in lung cells and NF-κB activity in monocytes [20].

Therefore, CBD influences numerous skin processes, particularly through its antioxidant and anti-inflammatory activities. These properties are relevant to aging and wound healing, making CBD an appealing natural ingredient for the development of cosmeceuticals.

Nevertheless, the safety concerns about CBD for the short and long term remain unclear, and some of its bioactivities in cosmetic application are still controversial, with a lack of cellular-level evidence to confirm its topical benefits. Thus, this study aimed to investigate the safety and potential bioactivities of CBD by focusing on relevant biomarkers, especially regarding anti-aging and wound healing activities, at the cellular level for cosmeceutical application.

## 2. Results

### 2.1. The Cytotoxic Effect of Cannabidiol on Fibroblasts and Keratinocytes

To evaluate the effect of CBD on short-term cytotoxicity, an SRB assay was used for evaluation in this study. The fibroblasts and keratinocytes were treated with CBD in concentrations in the range of 0–40 µg/mL for 24 h.

**Figure 1:**
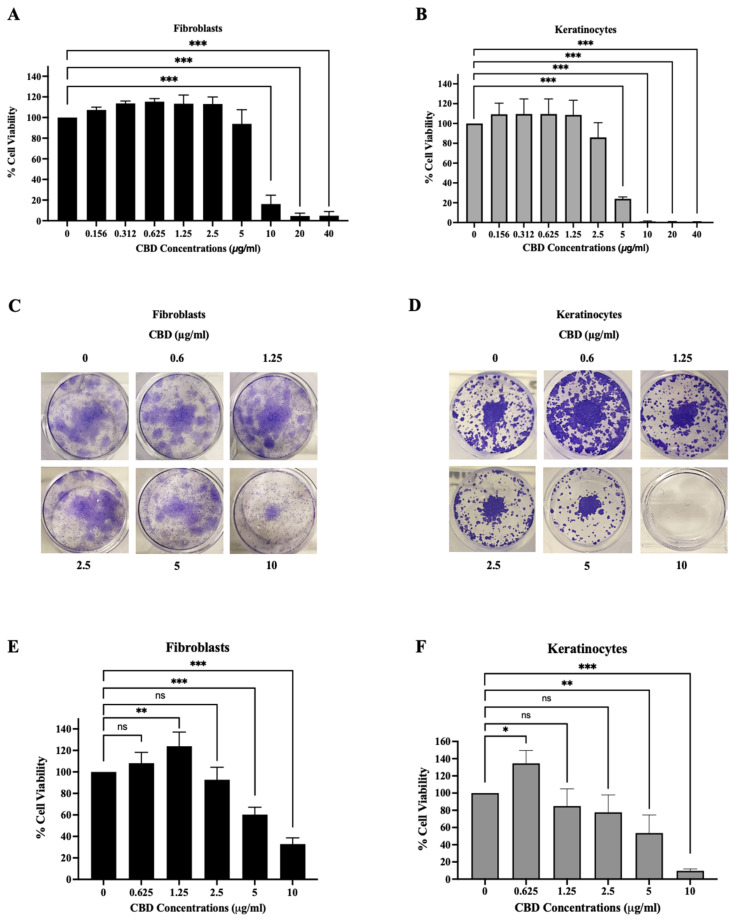
The short-term cytotoxicity of cannabidiol (CBD) at concentrations of 0–40 µg/mL on (**A**) fibroblasts and (**B**) keratinocytes. Images of the long-term effects on (**C**) fibroblasts and (**D**) keratinocytes after treatment with CBD for 24 h and after 10 days; we calculated the relative cell viability from the clonogenic assay of (**E**) fibroblasts and (**F**) keratinocytes in comparison with the control group. The data are presented as the mean ± and standard deviation (±SD) from three independent experiments (*n* = 3). Statistical significance was determined by a one-way ANOVA (non-significant; ns, significant; * *p* < 0.05, ** *p* < 0.01, and *** *p* < 0.001 vs. control (0 µg/mL)).

The results show that the concentrations of CBD ranging from 0.156 to 2.5 µg/mL were not toxic to fibroblasts (Figure 1A) and keratinocytes (Figure 1B) in the short-term timepoint, as indicated by the cell viability remaining above 70%, in accordance with ISO 10993-5, an international standard for evaluating the in vitro cytotoxicity of medical devices and materials [21]. However, at the concentration of 5 µg/mL, CBD only remained non-toxic to fibroblasts (Figure 1A). To further evaluate the safety of CBD on the skin, its impact on colony formation, and its effects after CBD withdrawal, a clonogenic assay was conducted. Keratinocytes and fibroblasts were treated with CBD at concentrations of 0, 0.625, 1.25, 2.5, 5, and 10 µg/mL for 24 h, and the long-term cytotoxicity was assessed 10 days post-treatment. Our results show that CBD at concentrations of 0.625–2.5 µg/mL was nontoxic to keratinocytes (Figure 1D) in the long term and could induce cell proliferation when activated at a very low concentration (0.625 μg/mL) (Figure 1F). Similarly, CBD at concentrations of 0.625–2.5 µg/mL was non-toxic to fibroblasts (Figure 1C). Notably, CBD at a concentration of 5 µg/mL was toxic to both fibroblasts and keratinocytes in the long-term clonogenic assay. We also observed that the concentration of CBD at 1.25 µg/mL could induce the cell proliferation of fibroblasts (Figure 1E). Interestingly, we found that CBD at low concentrations (0.625–2.5 μg/mL) did not adversely affect cell viability in either the short or long term in keratinocytes and fibroblasts, and the effects of CBD within this concentration range on skin cells appear to be reversible after 24 h of contact. However, CBD at concentrations exceeding 5 µg/mL seemed to result in irreversible effects on cells within a 10-day period. Thus, this concentration range was selected for further investigation into the potential biological effects of CBD in subsequent experiments.

### 2.2. The Effect of Cannabidiol on Antioxidant Activity in Fibroblasts and Keratinocytes

**Figure 2:**
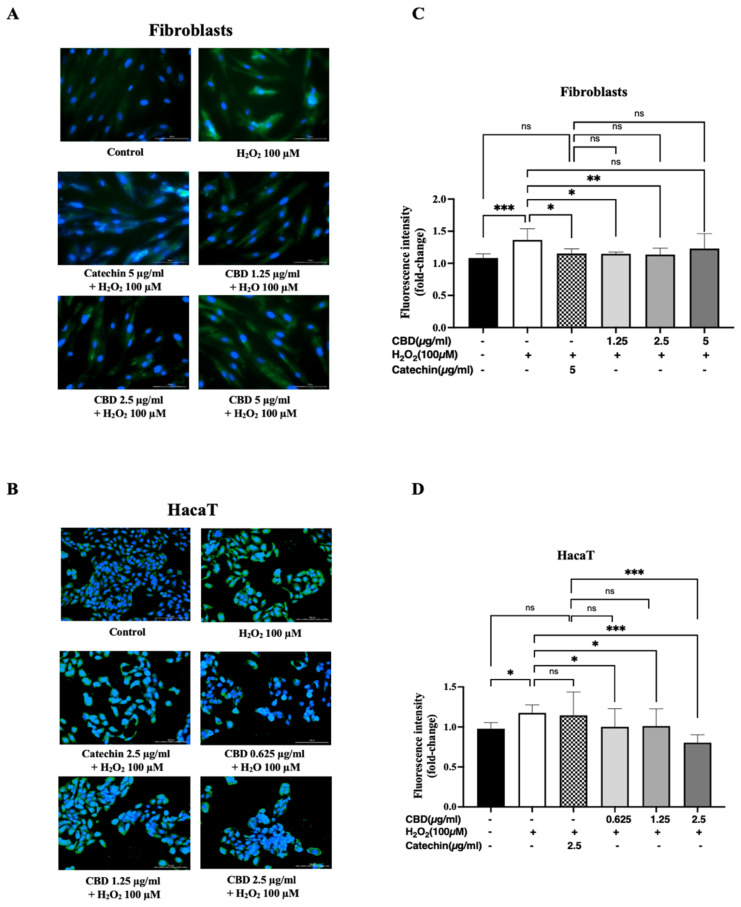
ROS production of CBD-treated cells before being activated with 100 µM of H_2_O_2_ for 24 h in (**A**) fibroblasts and (**B**) HaCaT keratinocytes. Green fluorescence (CellROX) represents intracellular ROS, and blue fluorescence (DAPI) represents cell nucleus. Relative fluorescence intensity after CellROX staining of (**C**) fibroblasts and (**D**) HaCaT keratinocytes. Green fluorescence intensity was normalized using DAPI fluorescence intensity as background. Data are presented as mean ± and standard deviation (±SD) from three independent experiments (*n* = 3). Statistical significance was determined by one-way ANOVA (non-significant; ns, significant; * *p* < 0.05, ** *p* < 0.01, and *** *p* < 0.001 vs. negative control (H_2_O_2_ 100 µM) and positive control (catechin 5 µg/mL)).

We aimed to evaluate the potential cellular bioactivities of cannabidiol for cosmeceutical application. The antioxidant activity of cannabidiol was measured from the intracellular ROS level after the cells were treated with CBD for 24 h and after the intracellular ROS level was activated by 100 µM H_2_O_2_ for 24 h. Our results indicate that CBD reduced the green fluorescence intensity in fibroblasts (Figure 2A,C) and keratinocytes (Figure 2B,D) when compared with the negative control (100 µM H_2_O_2_). In addition, CBD at the concentrations of 1.25 and 2.5 µg/mL could decrease oxidative stress in the cells to the same level as the positive control (catechin 5 µg/mL) in fibroblasts (Figure 2C). Like with keratinocytes, CBD at concentrations in the range of 0.625–2.5 µg/mL could decrease oxidative stress in the cells (Figure 2D).

### 2.3. The Effect of Cannabidiol on Anti-Aging Activity in Fibroblasts and Keratinocytes

**Figure 3:**
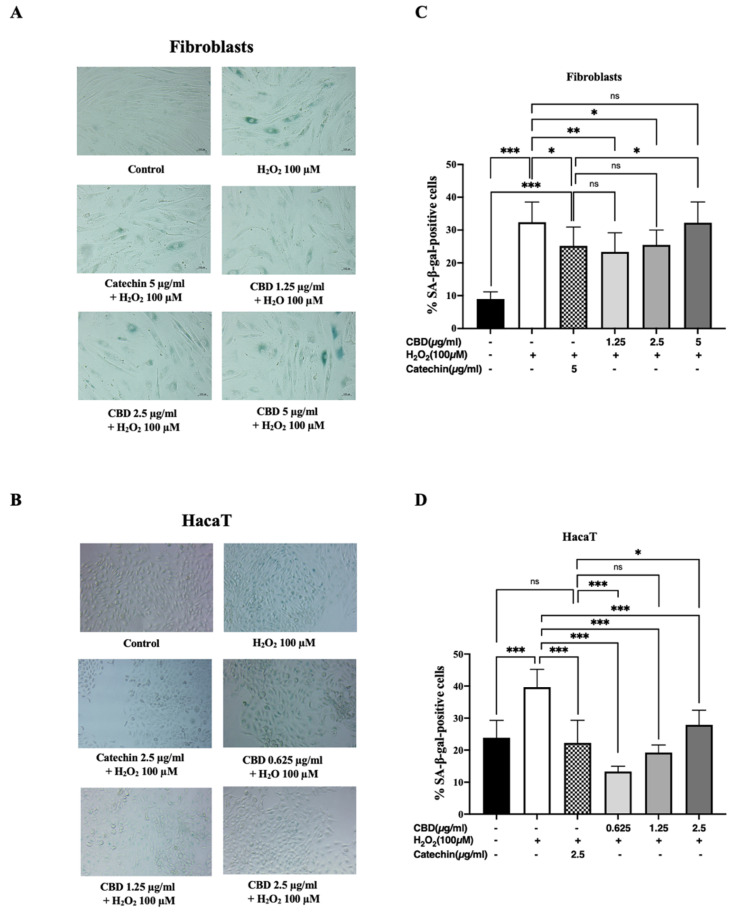
Senescence associated β-galactosidase (SAβgal) staining in (**A**) fibroblasts and (**B**) HaCaT keratinocytes after being pretreated with cannabidiol (CBD) before being stimulated by 100 µM of H_2_O_2_ for 24 h. The senescent cells are blue in color. The percentages of SA-β-gal-positive cells after being pretreated with CBD on (**C**) fibroblasts and (**D**) HaCaT keratinocytes. The data are presented as the mean ± and standard deviation (±SD) from three independent experiments (*n* = 3). Statistical significance was determined by a one-way ANOVA (non-significant; ns, significant; * *p* < 0.05, ** *p* < 0.01, and *** *p* < 0.001 vs. negative control (H_2_O_2_ 100 µM) and positive control (catechin 5 µg/mL)).

In a previous study, our results demonstrated that CBD could reduce oxidative stress in cells, which is linked to cell senescence. To investigate the effect of CBD on anti-aging activity, the senescent cells were measured by senescence β-galactosidase staining. Our results show that fibroblasts, after being pretreated with CBD and being stimulated with 100 µM of H_2_O_2_, exhibit less blue-colored cells (Figure 3A), as observed with keratinocytes (Figure 3B). Therefore, CBD at concentrations of 1.25 and 2.5 µg/mL could decrease % SA-β-gal-positive cells, as observed with the positive control (catechin 5 µg/mL) compared with the negative control (100 µM H_2_O_2_) in fibroblasts (Figure 3C). However, we observed that CBD at a concentration of 5 µg/mL could not reduce senescent cells, suggesting that higher doses of CBD may increase oxidative stress in cells, causing them to enter a senescent state similar to that triggered by 100 µM of H_2_O_2_. In keratinocytes, CBD at concentrations of 0.625–2.5 µg/mL could effectively decrease the % SA-β-gal-positive cells (Figure 3D), further confirming that CBD possesses the potential to reduce senescent cells due to oxidative stress.

### 2.4. The Effect of Cannabidiol on Wound Healing in Fibroblasts

**Figure 4:**
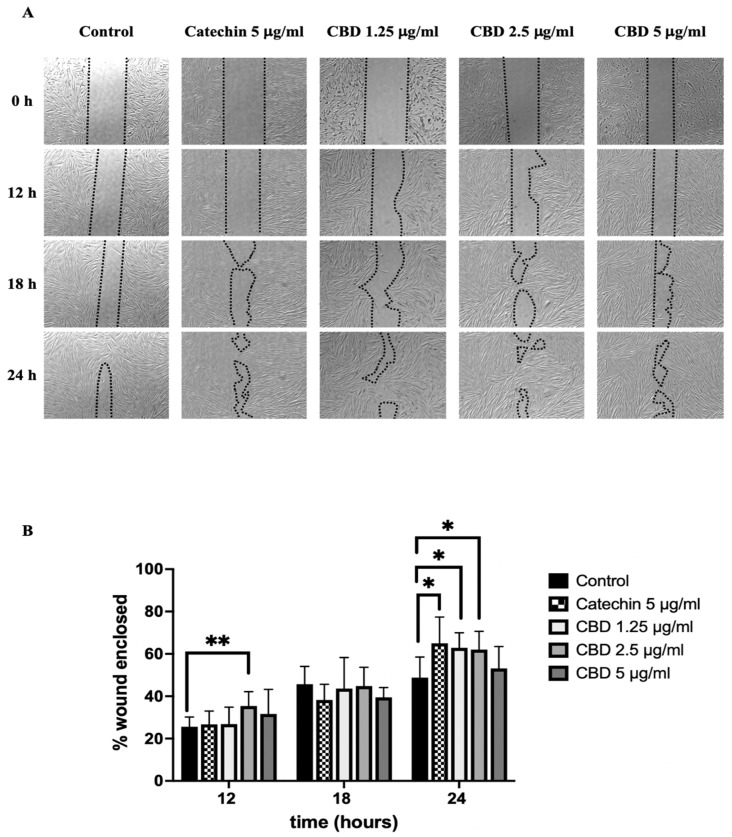
(**A**) Images of the wound healing assay of fibroblasts after forming gaps and being treated with CBD for 12, 18, and 24 h. (**B**) The bar graphs represent the percentages of wound-enclosed fibroblasts after being treated with CBD. The data are presented as the mean ± and standard deviation (±SD) from three independent experiments (*n* = 3). Statistical significance was determined by a one-way ANOVA (non-significant; ns, significant; * *p* < 0.05 and ** *p* < 0.01 vs. control (CBD 0 µg/mL)).

In a previous study, we found that CBD can enhance cell proliferation in the long term. Hence, the wound healing investigation was assessed by a wound healing assay. We formed a monolayer using fibroblasts and then created a wound gap before treating it with CBD (Figure 4A). Our results demonstrate that CBD at concentrations of 1.25 and 2.5 µg/mL can increase the %wound enclosed in fibroblasts within 24 h compared with the control (CBD 0 µg/mL) (Figure 4A,B). Moreover, CBD-treated cells can migrate to cell-free areas and form a bridge to close the gap after treatment with CBD (Figure 4A).

### 2.5. The Effect of Cannabidiol on Gene Expression Related to Skin Regeneration in Fibroblasts

**Figure 5:**
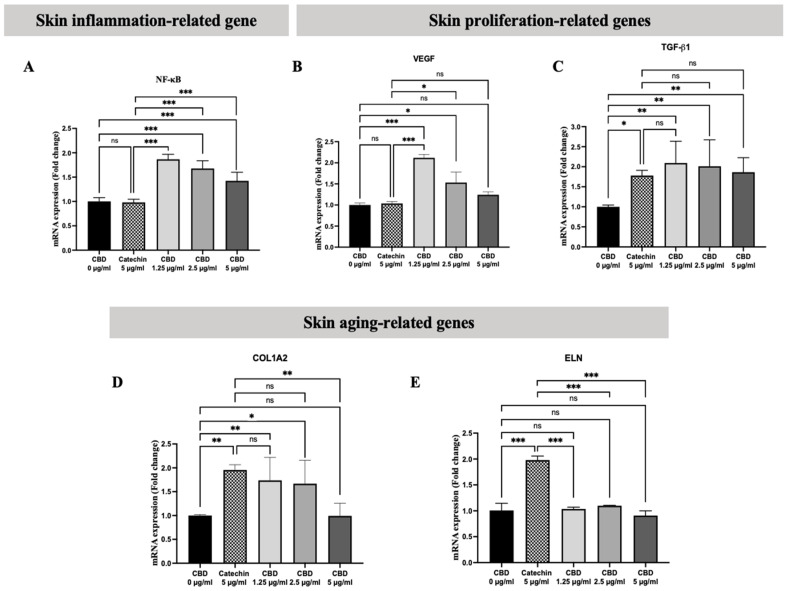
The mRNA expression of inflammation-related gene (**A**) NF-κB, proliferation-related genes (**B**) VEGF and (**C**) TGFB1, and skin aging-related genes (**D**) COL1A2 and (**E**) ELN after being treated with CBD in fibroblasts. The data are presented as the mean ± and standard deviation (±SD) from three independent experiments (*n* = 3). Statistical significance was determined by a one-way ANOVA (non-significant; ns, significant; * *p* < 0.05, ** *p* < 0.01, and *** *p* < 0.001 vs. control (CBD 0 µg/mL) and positive control (catechin 5 µg/mL)).

The bioactivity results of cannabidiol demonstrate that CBD has many bioactivities that can apply to cosmeceutical products. To identify the exact molecular mechanisms of CBD that are involved in skin regeneration, the expression of inflammatory genes such as NF-κB, proliferation genes such as TGF-β1 and VEGF, and skin aging genes such as COL1A2 and ELN was evaluated by qPCR. On fibroblasts, CBD showed activation effects on the mRNA expression levels of the CBD-treated cells by inducing NF-κB when compared with the gold standard (catechin) (Figure 5A). Moreover, CBD increased cell proliferation-related genes such as VEGF and TGF-β1 (Figure 5B,C). Additionally, we found that CBD at concentrations of 1.25–2.5 µg/mL could enhance the expression of COL1A2, which is related to collagen production (Figure 5D), and CBD did not affect the expression of ELN (Figure 5E). Therefore, our results confirm that CBD enhanced the expression of genes with skin proliferation potential and induced collagen production genes in fibroblasts.

## 3. Discussion

Cannabidiol (CBD) has become a compound used to develop extensive cosmeceutical commercial products. However, the safety and the unclear bioactivity of CBD are still concerns and need to be examined.

In this study, we first investigated the safety of CBD using both short-term and long-term in vitro assays. Previous studies reported that CBD at low concentrations (1–10 μM) had no effect on cytotoxicity in many cell lines, such as human epidermal melanocytes [22], THP-1-derived macrophages [23], and keratinocytes [16], in a short-term period. Similarly, our findings show that CBD at concentrations of 0.156–5 μg/mL was not toxic to fibroblasts, while CBD at concentrations of 0.156–2.5 μg/mL did not affect keratinocytes in short-term treatment (24 h). Additionally, the long-term cytotoxicity and colony-forming ability after CBD treatment for 10 days were evaluated using a clonogenic assay. The results demonstrate that very low CBD concentrations (0.6 and 1.25 µg/mL) did not induce cytotoxicity, but these concentrations significantly enhanced the growth of keratinocytes and fibroblasts, respectively. This is consistent with a previous study on *C. elegans* regarding long-term toxicity and lifespan, which found that CBD at concentrations of 10, 40, and 100 μM did not only show any toxicity to *C. elegans* but also improved late-stage life activity and extended the mean lifespan of *C. elegans* [24]. These results support our findings that CBD might have the promising potential to induce cell proliferation and promote some cell activities in the long term. Accordingly, we suggest that CBD at concentrations of 0.625–2.5 µg/mL is safe to use for skin applications.

However, we observed differences in significant effects on long-term cell growth after CBD treatment at a concentration of 1.25 µg/mL for 24 h between fibroblasts and keratinocytes. These differences likely reflect their distinct biological roles. These differences may arise from variations in CB1 and CB2 receptor availability and the activation of feedback mechanisms that influence the cellular uptake of CBD and sensitivity in these cell types [25]. Fibroblasts, which are primarily responsible for extracellular matrix production and tissue remodeling, may exhibit a more robust response to this CBD concentration compared to keratinocytes, whose primary function is to serve as a protective barrier. These functional distinctions could explain the variations in growth responses.

The absence of significant effects on long-term cell growth with 2.5 µg/mL of CBD treatment suggests that the 24 h treatment window may not be ideal for capturing dose-dependent effects. Future investigations should include extended timepoints (e.g., 48 and 72 h) to better understand these dynamics. Such experiments can determine whether the effects of CBD doses are delayed or not.

To develop cosmeceuticals using CBD, its antioxidant activity was first evaluated to assess its ability to scavenge ROS. Antioxidants protect cells from oxidative stress by donating electrons to free radicals, thereby neutralizing them and preventing cellular damage [26,27]. ROS are produced naturally by cells during metabolism and are also triggered by external factors like UV radiation and pollution. High ROS levels cause oxidative stress, which damages cell components and disrupts their function. This leads to cell aging and triggers inflammation [28,29]. In general, the skin has its own defense system to counteract ROS, comprising enzymatic antioxidants such as superoxide dismutase (SOD), catalase (CAT), glutathione peroxidase (GSH-Px), and glutathione reductase (GSH-Re), as well as non-enzymatic antioxidants such as vitamins C and E, and plant-derived compounds like catechins, curcumin, and resveratrol [30].

Therefore, in cosmeceutical research, assessing intracellular ROS levels is a fundamental step to understand the oxidative stress mechanisms that contribute to skin damage. This evaluation directly supports the practical use of antioxidants as skin-protective agents. Reducing ROS levels is also related to other beneficial bioactivities, such as anti-aging, anti-inflammatory, and photoprotective effects [30]. Therefore, measuring intracellular ROS levels is a critical step in gaining valuable insights into the mechanisms of skin damage and protection. This knowledge allows for the development of targeted formulations that inhibit specific signaling cascades involved in oxidative stress responses. Ultimately, linking ROS evaluation to skin protection mechanisms not only improves the scientific relevance of cosmeceuticals but also enhances their market value.

Interestingly, our study found that the pretreatment of skin cells with CBD at concentrations of 0.625–2.5 µg/mL could reduce the oxidative stress stimulated by 100 µM of H_2_O_2_ in fibroblasts and keratinocytes. Similarly, several studies previously reported that CBD protected the oxidative stress effect in the cells by reducing the total and mitochondria-generated ROS in cells [16] and by decreasing malondialdehyde (MDA) or 4-hydroxynonenal (4-HNE), which are related to the lipid peroxidation process in keratinocytes [31]. In addition, CBD is highly stable 24 h after exposure to UVA radiation with no phototoxicity in both primary human dermal fibroblasts and human keratinocytes, demonstrating that CBD can provide cytoprotection from UVA radiation [32]. According to all evidence, CBD has antioxidant activity and can decrease oxidative stress in skin cells.

Next, the anti-aging activity of CBD was assessed. Previous reports found that CBD failed to eliminate free radicals in defensive mechanisms, especially at the mitochondria, which leads to cellular aging [26,33,34]. In contrast, our results demonstrate that CBD at concentrations of 0.625–2.5 µg/mL could effectively reduce oxidative stress and decrease the percentage of SA-β-gal-positive cells after being treated with 100 µM of H_2_O_2_ for 24 h.

This finding can be further supported by previous studies of the lifespan and longevity of zebrafish (Danio rerio), which showed that the expression markers of senescence and inflammation were reduced after CBD treatment [35]. CBD improved the health span of *C. elegans* by modulating neuronal aging through the SIRT1/sir-2.1-autophagy pathway [36] and prevented neurite degeneration by regulating CB1-pSTAT3 signaling [37]. Furthermore, the combination of triacetylresveratrol and cannabidiol increased cell viability and wound healing functional activity in fibroblast cells and remedied nuclear eccentricity in senescent fibroblasts [38]. Correspondingly, this can confirm that CBD might have anti-aging potential.

Besides antioxidant and aging activities, the wound healing activity of CBD was also analyzed in fibroblasts. Proliferation is a process that increases the cell number by cell division, which is a crucial role of cell regeneration to repair cell damage. Generally, the wound healing process can be separated into three main phases: (i) the inflammatory phase; (ii) proliferative phase, and (iii) tissue remodeling phase [39,40]. Wound healing in the early stage starts with hemostasis and the activation of inflammatory cells to eliminate pathogens from the system and release pro-inflammatory cytokines, such as TNF-α, IL-6, and IL-1 β, which can activate keratinocytes, fibroblasts, and other cells [41]. Moving to the proliferative phase, keratinocytes and fibroblasts are activated by growth factors such as TGF-β1 and VEGF to activate the migration and proliferation of cells and induce new blood vessel formation for re-epithelialization [40]. The last stage is tissue remodeling, where fibroblasts synthesize collagen I to increase the tensile strength of the skin. After that, cell proliferation is decreased, and collagen is degraded by matrix metalloproteinases (MMPs), leading to the reorganization of the ECM to prevent scars and keloids [39,42]. Our results reveal that CBD at concentrations of 1.25 and 2.5 µg/mL can significantly improve the wound healing process in fibroblasts compared with the control. This finding can be supported by previous studies performed at the tissue level, which found that CBD can display strong regenerative results in both healthy and senescent injured tissues [38]. However, our study is a preliminary investigation of wound healing in fibroblasts, representing cells in the dermis layer of the skin, as they have a critical role in extracellular matrix remodeling and migration, which are key processes in the early phases of wound healing [43]. For future studies, wound healing activity should be further explored in keratinocytes or in a layered skin architecture [44] to confirm the most suitable dose of CBD for real-world skin applications.

In the final process of this study, we explored the molecular mechanisms underlying the proposed CBD bioactivities on skin cells by testing the expression of key genes associated with these activities, including NF-κB, TGF-β1, VEGF, COL1A2, and ELN. Our results reveal that CBD at concentrations of 1.25–2.5 µg/mL induced the expression of both VEGF and TGF-β1. Additionally, CBD at the same concentrations promoted the expression of COL1A2, which is linked to collagen production in fibroblasts, and increased NF-κB expression.

TGF-β1 and VEGF are growth factors that promote cell migration and proliferation and induce new blood vessel formation, which is critical during the proliferation and remodeling stages of wound healing [45,46]. In the early phase of wound healing, TGFβ1 is rapidly upregulated and secreted by keratinocytes, platelets, monocytes, macrophages, and fibroblasts near the wound site. TGF-β1 plays a role in the migration of epithelial sheets at the wound edges by regulating integrins and activating PI3K. It also promotes fibroblast proliferation and the production of bioactive factors, such as collagen, fibronectin, MMPs, TIMPs, and PAI-1, which are crucial for the deposition and remodeling of the wound’s extracellular matrix (ECM). Additionally, TGFβ1 stimulates wound contraction through the induction of smooth muscle alpha-actin expression in fibroblasts and the induction of myofibroblast differentiation. Moreover, TGF-β-induced angiogenesis involves the induction of VEGF in both epithelial cells and fibroblasts [47,48].

VEGF, another key factor in wound healing, plays a pivotal role in angiogenesis. Studies on human wounds and animal models have shown that VEGF is produced early in the wound healing process and during its later stages. It also plays a significant role in repairing the epidermal barrier and the underlying dermis during the proliferation phase [49].

NF-κB is a transcription factor that is crucial for the inflammatory phase of wound healing in the skin [50], and it plays a role in regulating VEGF induction during the early proliferative phase [51]. Furthermore, NF-κB is known to influence various cellular processes, including growth, inflammation, differentiation, and apoptosis, and it is involved in the production of pro-inflammatory cytokines and chemokines [52]. Previous studies have shown that NF-κB promotes mammary epithelial proliferation [53], and NF-κB activation is necessary for the repair of damaged muscles of mesoangioblasts [54]. Additionally, some studies reported that the activation of NF-κB signaling can promote the wound healing process in many cells, such as NIH3T3 cells [55], non-transformed rat intestinal epithelial (RIE-1) cells [56], keratinocyte HaCaT [57], and fibroblast Hs68 cells [57]. Nevertheless, many studies reported that CBD can reduce NF-κB activity and decrease pro-inflammatory cytokine release [19,20,23]. Therefore, we suggested that pro-inflammatory cytokines should be studied to confirm the effect of CBD on cellular inflammation.

Furthermore, the increased expression of COL1A2 in fibroblasts after CBD treatment confirms that CBD has anti-aging potential. The reduction in collagen and elastin in the dermis layer is one of the signs of skin aging outcomes [58]. The production of collagen and elastin is linked to dermal fibroblasts; the reduction in collagen production thus contributes to the formation of wrinkles due to the weakened connection between the dermis and epidermis skin layers [59,60]. In summary, our study indicates that CBD influences the expression of key genes involved in all phases of the wound healing process, supporting its potential as a therapeutic agent for skin repair and anti-aging applications.

However, ensuring the safety of CBD is a top priority before incorporating it into cosmetic products. We recommend conducting more experiments in further studies, such as genotoxicity tests, because CBD can activate mediators (VEGF and TGF-β), which are necessary for angiogenesis and can potentially influence cancer growth. Although previous studies have reported that hemp extract does not exhibit genotoxicity in bacterial reverse mutation assays (Ames assay) [61,62], the safety and risks associated with long-term exposure to CBD should also be thoroughly confirmed.

Although our results suggest that CBD concentrations ranging from 0.6 to 2.5 µg/mL are safe for use at the cellular level, previous studies have reported that CBD is unstable and can be degraded by approximately 10% within 24 h under a temperature of 37 °C and pH 7.4 [63]. This degradation may lead to the formation of CBD oxidation products, which can affect the optimal concentrations required for cell culture testing in fibroblasts and keratinocytes. In this study, cells were treated with CBD in complete media (containing FBS) at 37 °C for 24 h to mimic the real body environment; under these conditions, some degree of CBD degradation and potential chemical interactions with growth factors might have occurred. Further confirmation tests are needed to evaluate these effects.

In addition to temperature, CBD has been reported to degrade under exposure to light, extreme temperatures, and acidic or basic conditions. To enhance the stability of CBD and reduce its degradation in cosmetic products, it should be protected from light and oxygen, stored at low temperatures, and formulated at a neutral pH [64,65,66]. Furthermore, studies have shown that CBD does not exhibit cytotoxicity, mutagenicity, or skin sensitization and have verified the absence of primary irritability, accumulated irritability, phototoxicity, and photosensitization [67]. Together with our results, these findings strongly support the notion that CBD can be a promising candidate for topical cosmetic applications. However, the efficiency of CBD when applied to real skin may be reduced due to factors such as the skin barrier, UV light exposure, and off-target binding, which lead to CBD degradation, reducing its potency and altering its bioactive properties before reaching the target layer. Therefore, the encapsulation of CBD may be a more effective delivery method as it can target cells directly and help control the degradation rate due to environmental factors.

Overall, our study demonstrated that CBD at low concentrations (0.625–2.5 µg/mL) was non-toxic to skin cells and that it holds promising potential for topical skin application. Our findings indicate that CBD exhibits notable antioxidative activity by reducing H₂O₂-induced ROS production in dermal fibroblasts. However, this contrasts with the results reported by Luz-Veiga, M. et al. (2024), who demonstrated that CBD exhibited low antioxidant capacity and was unable to inhibit ROS production induced by Urban Particulate Matter (UPM) exposure in HaCaT cells [67]. This discrepancy might be attributed to the fact that different CBD concentrations and different external stimuli can activate distinct defense mechanisms in different cell types. Additionally, we found that CBD regulates the expressions of many important genes involved in anti-inflammatory responses (e.g., NF-κB), collagen and elastin production (COL1A2 and ELN), and skin proliferation (VEGF and TGF-β1) in fibroblast cells at basal levels, reflecting its potential to be used in skin treatment applications. Our results partially agree with the study of Chen, X et al., 2023, which demonstrated that CBD displayed UV-induced skin damage protection by enhancing the expressions of COL1A and ELN [68].

Notably, our results also reveal that CBD significantly inhibits the formation of senescence-associated β-galactosidase in fibroblasts, a key hallmark of aging, and enhances wound healing by accelerating fibroblast wound closure. Therefore, our findings highlight the potential of CBD as a promising phytochemical ingredient for the development of anti-aging cosmeceutical products and wound management applications.

However, our study is a preliminary study that aimed to find the potential bioactivity of CBD on skin cells in a monolayer model, which may not reflect the complexity of the actual skin layer. For future studies, we recommend utilizing more complex models that better mimic the real skin environment, such as 3D skin models, ex vivo skin, or in vivo models. These models will be more effective tools for understanding the skin processes and safety after CBD application, leading to the confirmation of its safety and efficacy over a longer period of treatment.

## 4. Materials and Methods

### 4.1. Cells and Reagents

HaCaT keratinocytes and human dermal fibroblast (HDF) cell lines were purchased from ATCC (Manassas, VA, USA). Dulbecco’s Modified Eagle’s Medium (DMEM), fetal bovine serum (FBS), GlutaMAX, and penicillin/streptomycin were purchased from Gibco (Life Technologies Ltd., Paisley, UK). Cannabidiol (CBD) solution (1000 ug/mL in methanol) was purchased from Dr. Ehrenstorfer (LGC Labor GmbH, Augsburg, Germany). DAPI staining and the CellROX™ Green Reagent Kit were purchased from Invitrogen™ (Thermo Fisher Scientific, Waltham, MA, USA). The senescence β-galactosidase staining kit was purchased from Cell Signaling Technology (Cell Signaling Technology, Inc., Beverley, MA, USA). RNeasy Mini Kit was purchased from Qiagen (Qiagen, Hilden, Germany). Superscript III reverse transcriptase kit and oligo-dT primers were purchased from Invitrogen (Invitrogen, Paisley, UK). The primers were synthesized from Sigma-Aldrich (Merck KGaA, Darmstadt, Germany). HOT FIREPol^®^ EvaGreen^®^ qPCR Mix Plus (no ROX) was purchased from Solis Biodyne (Solis Biodyne, Tartu, Estonia).

### 4.2. Cell Culture

HaCaT keratinocytes and human dermal fibroblast (HDF) cell lines were cultured in Dulbecco’s Modified Eagle’s Medium (DMEM) and supplemented with 10% fetal bovine serum (FBS), GlutaMAX, and 1% penicillin/streptomycin. Cells were incubated at 37 °C with 5% CO_2_ and 95% humidity. Cell media were replenished every 3 days, and cells were subcultured when the cells had 70% cell confluency.

### 4.3. Short-Term Cytotoxicity

In the cytotoxicity study, we evaluated the toxic effects of CBD after 24 h of exposure to the cells. For the short-term cytotoxicity study, cytotoxicity was measured immediately after 24 h of CBD exposure using the SRB assay.

HaCaT keratinocytes and human dermal fibroblasts (HDFs) were seeded on a 96-well plate. For the preparation of the CBD solution used in the in vitro experiments, CBD was diluted in Dulbecco’s Modified Eagle’s Medium (DMEM) and supplemented with 10% fetal bovine serum (FBS), GlutaMAX, and 1% penicillin/streptomycin. Then, the cells were treated with CBD in varied concentrations (0–40 µg/mL) for 24 h. The cells were fixed with 10% trichloroacetic acid (TCA) (Sigma-Aldrich, St Louis, MO, USA) for 1 h. The cells were washed with deionized water, and 0.4% SRB solution (Sigma-Aldrich, MO, USA) was added to a 96-well plate and incubated for 30 min. The cells were rinsed with 1% acetic acid (QRëC™, New Zealand), and bound SRB was solubilized by 10 mM Tris Base (Loba Chemie, Mumbai, India). The absorbance was measured by using a spectrophotometric microplate reader (Thermo Fisher Scientific, Waltham, MA, USA) at a wavelength of 564 nm and calculating the percentage of cell viability.

### 4.4. Long-Term Cytotoxicity (Clonogenic Assay)

In the long-term cytotoxicity study, cells were treated with CBD for 24 h, after which the CBD was removed, and fresh complete media were added. The cells were then incubated for 10 days before cytotoxicity was assessed using the clonogenic assay. Briefly, the cells were seeded in 24-well culture plates. CBD in varied concentrations (0–10 µg/mL) was added and incubated for 24 h. The medium containing CBD was removed and changed to normal medium. Fresh medium was replenished every 2–3 days for 10 days. Then, the cells were fixed with 4% paraformaldehyde (Sigma-Aldrich, St Louis, MO, USA) for 20 min, and cells were stained with crystal violet solution (Sigma-Aldrich, St Louis, MO, USA) for 1 h. Running tap water was added to wash the cells, and they were dried at room temperature overnight. Finally, the stained colonies were dissolved in 10% acetic acid (QRëC™, New Zealand), and the absorbance was measured at a wavelength of 590 nm by using microplate readers (Thermo Fisher Scientific, Waltham, MA, USA).

### 4.5. Measurement of Intracellular ROS

Human dermal fibroblast (HDF) cells and HaCaT keratinocytes were seeded in a 12-well plate for 24 h. Cells were pretreated with CBD in varied concentrations (0–5 µg/mL) for 24 h and then stimulated with 100 µM H_2_O_2_ (Sigma-Aldrich, Darmstadt, Germany) for 24 h. Then, cells were fixed with 4% formaldehyde (Sigma-Aldrich, St Louis, MO, USA) at room temperature for 20 min and washed 3 times with PBS (Merck, Rockland, MA, USA). Cells were permeabilized by 1% TritonX (Sigma-Aldrich, St Louis, MO, USA) 100 for 10 min. After that, cell nuclei and the intracellular ROS were observed by DAPI staining and CellROX™ Green Reagent Kit (Invitrogen, Carlsbad, CA, USA) by following the manufacturer’s instructions. The green fluorescence signal in cells was observed using a Lionheart FX Automated Microscope (Biotek, Winooski, VT, USA), and the fluorescent intensity was measured. The quantitative intensity data were collected from images by image statistics from Gen5 software version 2.0 (Biotek, Winooski, VT, USA). The total fluorescent intensity was calculated by the sum of pixel intensities above or below the threshold. Green fluorescence intensity was normalized using DAPI fluorescence intensity as a background. The data were collected from 3 independent experiments, with triplicate fields in each experiment, with the relative total fluorescent intensity being presented as the mean ± standard deviation (±SD) (*n* = 3).

### 4.6. Cellular Senescence Detection

HDFs cells and HaCaT keratinocytes at 1.5 × 10^5^ cells/well were cultured in a 6-well plate for 24 h, pretreated with CBD in varied concentrations (0–5 µg/mL) for 24 h, and then stimulated with 100 µM H_2_O_2_ (Sigma-Aldrich, Darmstadt, Germany) for 24 h. Then, the medium was removed, and a fresh complete medium was added to a 6-well plate. Cells were incubated at 37 °C with 5% CO_2_ for 3 days. The senescent cells were detected using the senescence β-galactosidase staining kit (Cell Signaling Technology, Beverley, MA, USA). Then, 1 mL of the β-galactosidase staining solution was added, and the cells were incubated at 37 °C without CO_2_ overnight. The senescent cells (blue stained cells) were observed using a microscope, and the percentage of senescent cells was calculated. The data were collected from 3 independent experiments, with triplicate fields in each experiment, with the percentages of SA-β-gal-positive cells being presented as the mean ± standard deviation (±SD) (*n* = 3).

### 4.7. Wound Healing Assay

The total of HDFs cells 1.5 × 105 cells/well were cultured in a 12-well plate until a monolayer was formed. Then, cells were starved for 24 h by media containing 1%FBS. Cells were scratched with a 200 μL pipette tip to create the gap. After that, the cells were treated with CBD in varied concentrations (0–5 µg/mL) for 24 h, and pictures were captured every 6 h for 24 h using a Lionheart FX Automated Microscope (Biotek, Winooski, VT, USA). The picture of the wound was analyzed by using Gen5 software (Biotek, Winooski, VT, USA) to calculate the percentage of wounds enclosed compared with the control group.

### 4.8. Real-Time Quantitative PCR (qPCR)

The total cell RNA was extracted from cell pellets using an RNeasy Mini Kit (Qiagen, Hilden, Germany). cDNA was generated by using a Superscript III reverse transcriptase kit and oligo-dT primers (Invitrogen, Paisley, UK) following the manufacturer’s instructions. A real-time analysis was performed on a CFX connect real-time system (Bio-Rad, Hercules, CA, USA). All primers were validated to avoid secondary structures, primer dimers, or complementary sequences that may reduce specificity by bioinformatics tools (e.g., Primer-BLAST, Oligocalc). The primer obtained was amplified with HOT FIREPol^®^ EvaGreen^®^ qPCR Mix Plus (no ROX) (Solis Biodyne, Tartu, Estonia). All of the experiments had a No Template Control (NTC) in every condition to detect contamination or non-specific amplification in each independent experiment. The results were normalized using the ribosomal protein L19 mRNA expression as a reference gene. All qPCR experiments were conducted from triplicate samples of three independent experiments, with the relative expression being presented as the mean ± standard deviation (±SD) (*n* = 3). The primer sequences are described in Table 1.

### 4.9. Statistical Analysis

All data were presented as the mean ± and standard deviation (±SD) for at least three independent experiments (*n* = 3). Statistical significance was performed using GraphPad Prism version 9.4.1 (GraphPad Software, San Diego, CA, USA) and calculated using one-way ANOVA between control and treatment conditions for more than two groups. A *p*-value of ≤ 0.05 was considered as statistical significance between groups.

## 5. Conclusions

In conclusion, our study investigated the safety and potential bioactivities of CBD at the cellular level and explored its potential mechanisms for cosmeceutical application. Regarding CBD’s safety in the short term and long term, our results indicate that CBD at low concentrations is safe to be used on the skin and promotes the growth of both keratinocytes and fibroblasts. In terms of functional CBD bioactivity studies, we found that CBD showed promising antioxidant activity and anti-aging activity in fibroblasts and keratinocytes. In terms of involved molecular mechanisms, we found that CBD promoted TGF-β1, VEGF, and NF-κB expression in fibroblasts, which are related to proliferative and wound healing processes. In addition, CBD can increase collagen production in fibroblasts through CO1A2 expression. Overall, we suggest that CBD exhibits many potential characteristics that can be used to develop topical cosmeceutical products, such as sun protection products, hair care products, or wound healing products. For future studies, we recommend using 3D skin models, ex vivo skin, or in vivo models to better understand skin processes after CBD application and to confirm the safety and activities of CBD over a longer period of time.

## Figures and Tables

**Table 1:** Primer sequences for real-time quantitative PCR (qPCR).

Gene	Forward Primer (5′>3′)	Reverse Primer (5′>3′)
NF-κB	GGAATGGTGAGGTCACTCTA	AGAATGAAGGTGGATGATTG
ELN	CTGCAAAGGCAGCCAAATAC	CACCAGGAACTAACCCAAACT
COL1A2	GCAACCTGAAAAAGGCTGTC	GGCGTGATGGCTTATTTGTT
TGF- β1	GTCTGCTGAGGCTCAAGTTA	AGTGTGTTATCCCTGCTGTC
VEGF	CTTGCCTTGCTGCTCTACCT	CACACAGGATGGCTTGAAGA
L-19	GCGGAAGGGTACAGCCAAT	GCAGCCGGCGCAAA

## Data Availability

No data were used for the research described in this article.
